# Parapapillary atrophy and changes in the optic nerve head and posterior pole in high myopia

**DOI:** 10.1038/s41598-020-61485-2

**Published:** 2020-03-12

**Authors:** Mi Sun Sung, Hwan Heo, Helong Piao, Yue Guo, Sang Woo Park

**Affiliations:** Department of Ophthalmology and Research Institute of Medical Sciences, Chonnam National University Medical School and Hospital, Gwangju, South Korea

**Keywords:** Anatomy, Eye diseases

## Abstract

We investigated the relationship between microstructure of β-parapapillary atrophy (β-PPA) and morphologic features of optic nerve head (ONH) and posterior pole in highly myopic eyes. Eighty-nine highly myopic eyes were included in this study. Bruch’s membrane opening (BMO) area, lamina cribrosa (LC) thickness, anterior laminar depth, peripapillary and subfoveal choroidal thickness (CT), macular Bruch’s membrane (BM) length, and width of β-PPA with and without Bruch’s membrane (PPA_+BM_ and PPA_−BM_) were evaluated. The mean age and axial length of the included subjects were 26.88 ± 2.44 years and 27.03 ± 0.88 mm, respectively. The width of PPA_−BM_ was larger with increasing BMO area (*P* = 0.001), whereas the BMO area was not associated with the width of PPA_+BM_. The large PPA_+BM_ was significantly related to a thinner LC (*P* = 0.003), deeper anterior lamina surface (*P* < 0.001), longer macular BM length (*P* = 0.008), and thinner temporal peripapillary CT (*P* = 0.034). We found that the morphologic features of the ONH and posterior pole in highly myopic eyes were different based on the microstructure of β-PPA. Whether these features are linked to the development of glaucoma in myopic eyes should be investigated in future studies.

## Introduction

Classic funduscopic β-parapapillary atrophy (β-PPA) is a region of visible large choroidal vessels and sclera due to the lack of retinal pigment epithelium (RPE). β-PPA can be histologically subclassified based on the presence or absence of Bruch’s membrane (BM): β-PPA with BM (PPA_+BM_; β zone) and β-PPA without BM (PPA_−BM_; γ zone). It has been known that PPA_+BM_ might be due to age-related atrophy of the RPE and associated with the development of glaucoma^1–4^. Whereas, PPA_−BM_ has been considered as a result of mechanical stretching in the peripapillary sclera and border tissue during axial elongation; hence a significant association between axial length and PPA_−BM_ width has been consistently reported^1–3,5^. However, we previously reported that PPA_+BM_ was also found in 20–30-year-old myopia subjects and suggested that PPA_+BM_ in young myopia patients might be related to axial elongation, not age-related atrophy^6^. In accordance with our report, Lee *et al*.^7^ recently found an enlargement of PPA_+BM_ as myopia progressed and proposed that PPA_+BM_ found in children might be an entirely different entity from PPA_+BM_ in elderly individuals, sharing an etiology with PPA_−BM_.

Several factors might contribute to β-PPA development during axial elongation, but the etiology of PPA_+BM_ and PPA_−BM_ enlargement remains elusive so far. It is unclear why some myopia patients exhibit only PPA_−BM_, whereas others with a similar degree of myopia exhibit both PPA_+BM_ and PPA_−BM_. In this study, we hypothesized that morphologic features of the optic nerve head (ONH) and the posterior pole might give clues to the mechanisms of differential developments of β-PPA in myopic eyes. Therefore, the purpose of this work was to investigate the relationship between microstructure of β-PPA and the morphologic features of ONH and posterior pole in highly myopic eyes and to determine the mechanism of differential developments of β-PPA in myopic eyes.

## Methods

### Subjects

Subjects were recruited from the Young Myopia Study of Chonnam National University Hospital, an ongoing cross-sectional study that commenced in January 2018. The present study was conducted according to the tenets of the Declaration of Helsinki and was approved by the Institutional Review Board of Chonnam National University Hospital (CNUH-2018-147). All patients provided written informed consents before enrollment in the study.

The Young Myopia Study enrolled consecutive participants who visited the general eye clinic for medical check-up and met all of the inclusion criteria and none of the exclusion criteria. The details on the study protocol is also described in our recent report^8^. All subjects underwent comprehensive ophthalmologic examination consisting of the measurement of best-corrected visual acuity (BCVA), intraocular pressure (IOP) by Goldmann applanation tonometry, and refractive error by automated refraction. Anterior chamber angle assessment using gonioscopy was performed on all eyes. ONH and RNFL examination using color stereoscopic disc photography and red-free RNFL fundus photography, and Swedish Interactive Threshold Algorithm standard 30-2 perimetry with a Humphrey Field Analyzer (Carl Zeiss Meditec Inc., Dublin, CA, USA) were performed. Axial length, central corneal thickness, and corneal curvature were measured using optical low-coherence reflectometry (Lenstar; Haag-Streit AG, Koeniz, Switzerland). For all subjects, a detailed medical history was recorded.

The inclusion criteria were: (1) healthy subjects 20 to 35 years old, (2) highly myopic eyes with axial length greater than 26 mm, (3) astigmatism within ± 2 diopters (D), (4) BCVA of 20/25 or better, (5) IOP ≤ 21 mmHg, (6) normal anterior chamber angles, (7) nonglaucomatous ONHs on stereoscopic photographs (8) absence of any RNFL abnormalities on red-free fundus photographs, and (9) normal visual field (VF) results in both eyes. Because myopic refractive error can be affected by lenticular changes, and aging may increase the incidence of glaucoma, we excluded subjects older than 35 years. Normal VF presentation was defined as a glaucoma hemifield test result within normal limits, as well as mean and pattern standard deviation values associated with probabilities of normality higher than 5%. Some eyes had an enlarged blind spot associated with a large area of PPA, and such eyes were also included in this study.

Subjects were excluded if they had any of the following: (1) a family history of glaucoma in a first-degree relative, (2) history of intraocular or refractive surgery, (3) pathologic myopia (patch chorioretinal atrophy, lacquer crack lesions, intrachoroidal cavitations, or choroidal neovascularization), (4) other evidence of retinal pathology, (5) opaque media, or (6) poor-quality OCT images because of irregular tear film or poor cooperation.

Eligibility was determined by two glaucoma specialists (S.W.P and M.S.S). The evaluators were blinded to all other patient and ocular data, and an eye was excluded from the study if a consensus was not reached. In cases where both a subject’s eyes met the inclusion criteria, one eye was randomly selected for the study.

### Spectral-domain optical coherence tomography imaging

All participants underwent OCT imaging using SD-OCT (Heidelberg Spectralis SD-OCT; Spectralis software version 6.9.4; Heidelberg Engineering GmbH, Heidelberg, Germany). One experienced operator performed all the OCT scans. Magnification error was corrected using the formula provided by the manufacturer based on the results of keratometry and focus setting during image acquisition. OCT images with insufficient quality (typically truncated B-scans and quality score <30) were excluded.

**Figure 1:**
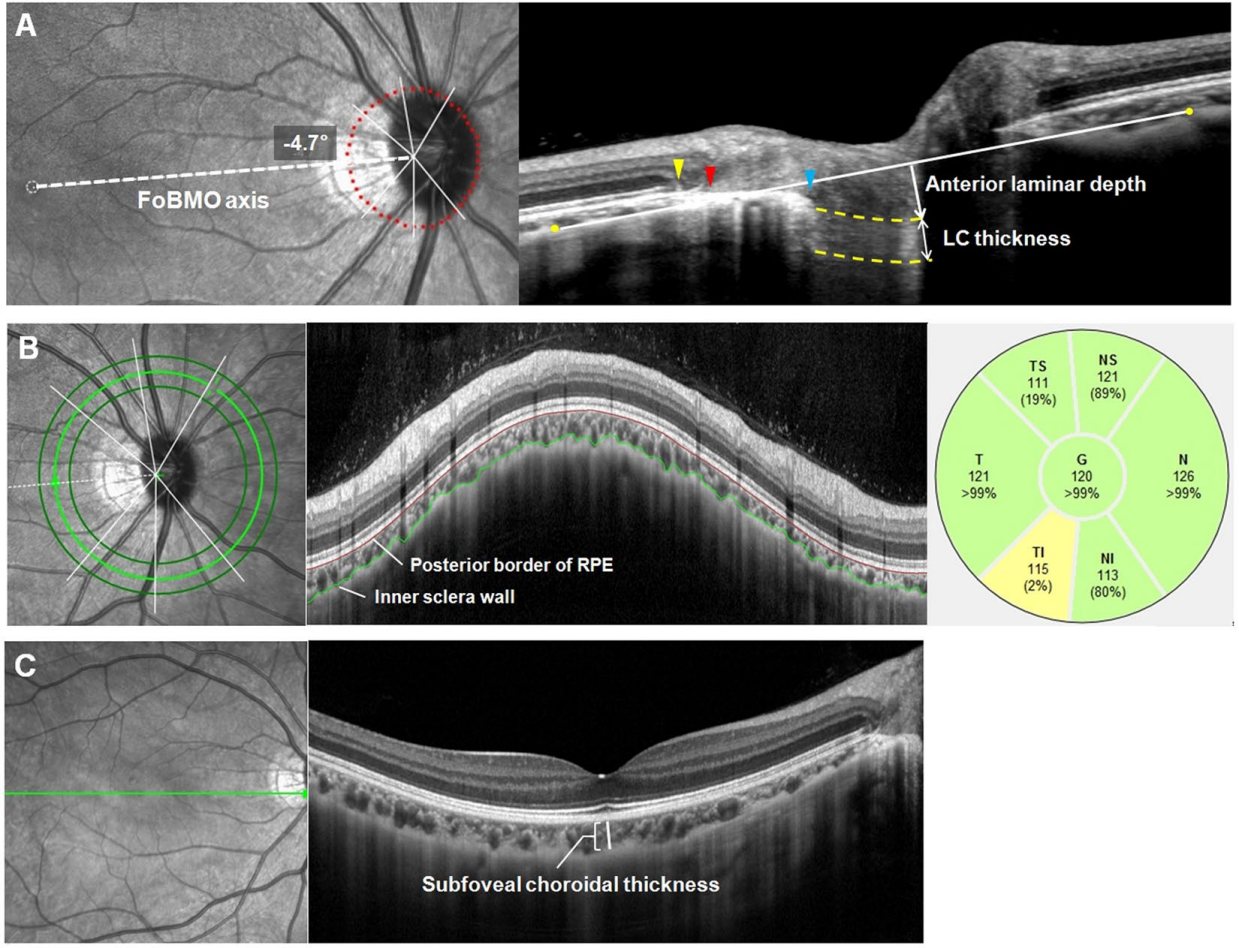
Methods used for the ONH and CT analysis by using SD-OCT. **(A)** Bruch’s membrane opening (*red dots*) is identified on confocal scanning laser ophthalmoscopy and B-scan images. FoBMO axis (the line connecting the center of the fovea and BMO center) and FoBMO angle are also illustrated on the fundus image. Scan from EDI OCT scan image passing through the ONH (center, midsuperior, and midinferior) is also presented. The *yellow arrow head* indicates the RPE termination, *red arrow head* indicates the termination of BM, and *blue arrow head* indicates the optic disc margin. The distance from the edge of BM (*red arrow head*) to the margin of RPE termination (*yellow arrow head*) and to the optic disc margin (*blue arrow head*) are defined as PPA_+BM_ width and PPA_−BM_ width, respectively. Anterior lamina depth is defined as the distance between the sclerochoroid junction reference line (*white solid line*) and the anterior border of the LC (*yellow dashed line*). LC thickness is defined as the distance between the anterior and posterior borders (*yellow dashed lines*) of the LC. **(B)** Circular peripapillary scan of 4.1 mm is obtained. After the manual segmentation of BM (*red solid line*) and the sclerochoroidal border (*green solid line*), peripapillary CT was calculated. The global and the 6 Garway-Heath regional values (NS, N, NI, TI, T, and TS) was used. **(C)** EDI horizontal scan passing through the center of the fovea was used to measure subfoveal CT. ONH = optic nerve head; CT = choroidal thickness; BM = Bruch’s membrane; BMO = Bruch’s membrane opening; EDI = enhanced depth imaging; RPE = retinal pigment epithelium; PPA_+BM_ = β-parapapillary atrophy with BM; PPA_−BM_ = β-parapapillary atrophy without BM; LC = lamina cribrosa.

Bruch’s membrane opening (BMO) area was measured using the Glaucoma Module Premium Edition (GMPE) software (Fig. 1A). The scan protocol composed of 24 equally spaced radial B-scans, each with 768 A-scans covering a 15° region centered on the optic disc. Twenty-five B-scans were averaged automatically for each scan location. The software automatically detected the 48 BMO points from the 24 radial scans. All the B-scans were manually checked, and BMO points were corrected when necessary. When BMO points were indiscernible, the points were fitted with a spline to derive a closed curve and a smooth contour line based on BMO points of adjacent B-scans^8^. If BMO points were indiscernible on four or more consecutive B-scan images, the eyes were excluded from the study. First, BMO points were checked by one experienced evaluator (M.S.S) and then reassessed and confirmed by a senior glaucoma specialist (S.W.P). In case of any discrepancy, the eyes were excluded from the analysis. BMO area was computed automatically by the built-in software. In this scan mode, the foveal pit and two BMO points in each of the two radial B-scans (that were perpendicular to each other) were automatically segmented to estimate the center of BMO and determine the FoBMO axis (the line connecting the center of the fovea and BMO center), which served as a reference for the scans. The FoBMO angle was defined as the angle between the FoBMO axis and the horizontal axis of the acquired image frame.

### Optic nerve head measurements

The Heidelberg Spectralis OCT enhanced depth imaging (EDI) mode was used for the other ONH measurements (Fig. 1A). The details on the ONH measurements are also described in our previous reports^6,8^. The ONH was scanned by centering a 15° × 10° rectangular scan on the ONH. Each OCT volume consisted of 49 serial horizontal B-scans (4.5 mm lines; 50 images averaged) spaced at approximately 63 μm intervals. Infrared (IR) fundus images were acquired simultaneously by using a confocal scanning laser ophthalmoscope. Three sections that passed through the ONH in the center, midsuperior, and midinferior regions were selected. Mid-superior and mid-inferior locations were defined as the middle of the vertical line connecting the center to the superior and inferior margin of the optic disc. The closest horizontal B-scan frames to mid-superior and mid-inferior points were chosen for image analysis. All the study parameters were measured in each of these frames by two independent, blinded examiners (M.S.S and H.H).

Temporal β-zone PPA margin and disc margin were defined using IR fundus images. Temporal BMO points were identified in the horizontal EDI scan image. PPA width was measured using the synchronous viewing of the IR fundus image and the selected location on the OCT scan. β-PPA width was defined as the distance between the beginning of the retinal pigment epithelium (RPE) (i.e., temporal β-PPA margin) and temporal disc margin on each horizontal B-scan image. Based on the location of BM termination, the β-PPA was further divided into PPA_+BM_ and PPA_−BM_. PPA_+BM_ width was defined as the distance from the beginning of the RPE to BMO, and PPA_−BM_ width was defined as the distance from the temporal disc margin to the beginning of BM. In case of the termination of BM is not clearly visible on the horizontal EDI B-scan, we reviewed the radial B-scans from GMPE mode to identify the result of BMO delineation, and referred to them for determination of BM termination points on the EDI B-scans. The measurement was performed using a previously described method^6–11^.

The sclerochoroidal junction reference plane was used to overcome the effect of choroidal thickness on lamina cribrosa (LC) depth measurements^12,13^. The sclerochoroidal junction reference line was defined as the line connecting two points of the anterior scleral surface located at 1750 μm from the center of BMO in each B-scan image. The vertical distance between the reference line and the anterior LC surface was measured at the center of the ONH and defined as anterior lamina depth. The anterior and posterior margins of the highly reflective region at the ONH vertical center in each B-scan image were used as the borders of the LC, and the perpendicular distance between these two borders was defined as LC thickness. In cases where the central retinal vessel trunk prevented visualization, measurements were performed on the temporal side.

The measurement was performed using a built-in caliper tool of the intrinsic OCT viewer, and average data of three horizontal B-scan images (center, midsuperior, and midinferior) were calculated and used in this study. The final analysis used the mean of the values obtained by the two examiners.

### Detection of lamina cribrosa defect

The EDI OCT images of the ONH were reviewed carefully for detecting focal LC defects. A focal LC defect was defined as a loss of high reflectivity from the anterior-to-posterior border of the full-thickness LC on B-scan images. To avoid false-positives, the defects were required to have had a maximal diameter greater than 100 μm and a depth greater than 30 μm, and to have been present in two adjacent B-scans^14,15^. Shadows were differentiated from LC defects on the basis of their characteristic signal void behind the vessels and tissues. LC defect margins were independent of the location of the vessels and neural tissues. Images were reviewed by 2 experienced glaucoma specialists (M.S.S and S.W.P), and disagreements were addressed via discussion between the 2 evaluators to achieve consensus. An eye was excluded from study analyses if a consensus could not be reached.

### Measurement of macular bruch’s membrane length

Using the scan running through the fovea and the center of the optic disc (from the posterior pole asymmetry analysis protocol of the Heidelberg Spectralis OCT), the macular BM lengths were measured^16^. Macular BM length was defined as the distance between the fovea to the end of BM, in the direction of the ONH^17^. The averaged data from two independent examiners (M.S.S and H.H) were used in this study.

### Choroid thickness measurement

We obtained 360° circular RNFL measurements centered on BMO center. Among the three circular scans along the peripapillary circles (diameters of 3.5, 4.1, and 4.7 mm), the 4.1 mm diameter scans were analyzed to minimize the interference of a large PPA on the OCT scan path. For the measurement of the peripapillary choroidal thickness (CT), the upper and lower segmentation lines of the circular scan were manually delineated. The lines were adjusted to align with the inner scleral wall and posterior border of the RPE to define the outer and inner boundaries of the choroid, respectively (Fig. 1B). Peripapillary CT was automatically computed using the RNFL thickness sector algorithms (global, nasal-superior [NS], nasal [N], nasal-inferior [NI], temporal-inferior [TI], temporal [T], and temporal-superior [TS]). Subfoveal CT was measured from the horizontal EDI scan running through the fovea. The vertical distance from the hyper-scattering outer border of the RPE to the inner border of the sclera at the fovea was defined as subfoveal CT (Fig. 1C)^8^. Images in which the RPE and chorioscleral interface could not be clearly identified were excluded from the analysis. The averaged data from two independent examiners (M.S.S and H.H) were used in this study.

### Statistical analysis

SPSS version 21.0 (SPSS, Chicago, IL, USA) was used for all statistical analyses. Agreement of PPA_+BM_ width, PPA_−BM_ width, LC thickness, anterior laminar depth, macular BM length, and peripapillary and subfoveal CT between two observers was assessed using the Bland-Altman method, which plots the means against differences^18^. The limits of agreement were defined as the mean differences of two measurements ± 1.96 standard deviations (SD) of the difference. The normality of distribution was verified using the Shapiro-Wilk normality test. Baseline characteristics were reported in counts and proportions or mean ± SD values as appropriate. Groups were compared using the chi-square test, Student’s t-test, or Mann-Whitney U test as appropriate. Linear regressions were used to search for associations between the microstructure of β-PPA and various ocular parameters. Standardized and unstandardized regression coefficients, with a 95% confidence level, were presented. When performing multivariate regression analysis, factors showing multicollinearity were not included as explanatory variables. All *P* values from linear regression analyses were adjusted to control the false discovery rate using the Benjamini-Hochberg procedure^19^. A *P* value of less than 0.05 was considered statistically significant.

## Results

Among the eyes enrolled in the Young Myopia Study, 101 eyes of 101 subjects met the inclusion criteria with axial lengths greater than 26 mm. Of these, seven eyes were excluded because of indiscernible BMO on four or more consecutive B-scan images or discrepancy in BMO determination between the glaucoma specialists (M.S.S and S.W.P). Five eyes were additionally excluded because of inadequate visualization of the anterior LC surface, posterior LC surface, or both. None of the subjects was excluded because of the inability to determine RPE and chorioscleral interface. Finally, the remaining 89 eyes of the 89 subjects with high myopia were evaluated. Among the 89 eyes, automated detection of BMO points was found to be inaccurate in 57 eyes (64.04%), and manual corrections were made. Interobserver agreement, determined using Bland-Altman plots, in the measurements of PPA_+BM_ width, PPA_−BM_ width, LC thickness, macular BM length, and peripapillary and subfoveal CT for all the subjects showed no systematic differences in measurements (data not shown). Demographic and ocular characteristics are described in Table 1. The mean axial length of included eyes was 27.03 ± 0.88 mm (range, 26.04–29.76 mm).

**Table 1:** Demographic characteristics of the highly myopic eyes.

Variables	Description
Number, n	89
Age (years)	26.88 ± 2.44
Male, n (%)	62 (69.66)
SE refractive error (D)	−6.91 ± 2.43
Axial length (mm)	27.03 ± 0.88
Central corneal thickness (μm)	552.61 ± 30.16
Corneal curvature (D)	42.50 ± 1.00
IOP (mmHg)	13.85 ± 2.31
BMO area (mm^2^)	2.68 ± 0.72
PPA_+BM_ width (μm)	236.69 ± 205.69
PPA_−BM_ width (μm)	360.87 ± 216.85
LC thickness (μm)	188.42 ± 30.61
Anterior laminar depth (μm)	329.89 ± 105.24
FoBMO angle (°)	−5.06 ± 3.80
Presence of LC defect, n (%)	38 (42.70)
Macular BM length (μm)	4065.02 ± 333.13
Subfoveal CT (μm)	231.65 ± 86.60
**Peripapillary CT (μm)**	
Global	144.19 ± 46.05
Temporal-superior	155.93 ± 55.54
Temporal	129.99 ± 58.57
Temporal-inferior	114.69 ± 44.94
Nasal-inferior	124.20 ± 40.96
Nasal	160.24 ± 47.05
Nasal-superior	170.10 ± 52.10

SE = spherical equivalent; D = diopters; IOP = intraocular pressure; BMO = Bruch’s membrane opening; PPA_+BM_ = β-parapapillary atrophy with Bruch’s membrane; PPA_−BM_ = β-parapapillary atrophy without Bruch’s membrane; LC = lamina cribrosa; FoBMO angle = angle of fovea-to-BMO-center axis relative to the horizontal axis of the image frame; BM = Bruch’s membrane; CT = choroidal thickness.

Data are expressed as mean ± standard deviation.

Table 2 shows the associations between the microstructure of β-PPA and ocular parameters after adjusting for age, sex, and axial length in highly myopic eyes. PPA_−BM_ was positively correlated with BMO area (*P* < 0.001), and negatively correlated with subfoveal, global peripapillary, temporal peripapillary, and temporal-inferior peripapillary CT (*P* = 0.005, *P* = 0.042, *P* = 0.004, and *P* = 0.021, respectively). Whereas, BMO area was not associated with the width of PPA_+BM_. The large PPA_+BM_ was significantly related to a thinner LC (*P* < 0.001), deeper anterior lamina surface (*P* = 0.002), larger FoBMO angle (*P* = 0.031), longer macular BM length (*P* < 0.001), and thinner subfoveal and temporal peripapillary CT (*P* = 0.009 and *P* = 0.005) in highly myopic eyes.

**Table 2:** Association between the microstructure of parapapillary atrophy and ocular parameters in highly myopic eyes.

Variables	PPA_+BM_ width			PPA_−BM_ width		
	Coefficients^*^	*P* value^*^	*P* value^†^	Coefficients^*^	*P* value^*^	*P* value^†^
Axial length (mm)	0.211	**0.026**	—	0.214	**0.044**	—
Central corneal thickness (μm)	−0.058	0.590	0.638	−0.166	0.121	0.134
Corneal curvature (D)	−0.060	0.607	0.619	0.067	0.563	0.506
IOP (mmHg)	0.068	0.536	0.475	0.071	0.519	0.456
BMO area (mm^2^)	0.227	0.082	0.107	0.448	**<0.001**	**<0.001**
LC thickness (μm)	−0.411	**<0.001**	**<0.001**	−0.115	0.282	0.283
Anterior laminar depth (μm)	0.312	**0.002**	**0.002**	0.155	0.147	0.197
FoBMO angle (°)	0.248	**0.012**	**0.031**	0.172	0.107	0.205
Macular BM length (μm)	0.352	**0.001**	**<0.001**	−0.040	0.708	0.848
Subfoveal CT (μm)	−0.345	**0.001**	**0.009**	−0.330	**0.001**	**0.005**
**Peripapillary CT (μm)**						
Global	−0.176	0.101	0.189	−0.240	**0.018**	**0.042**
Temporal−superior	−0.192	0.074	0.121	−0.179	0.080	0.133
Temporal	−0.331	**0.002**	**0.005**	−0.342	**0.001**	**0.004**
Temporal-inferior	−0.189	0.078	0.090	−0.286	**0.007**	**0.021**
Nasal-inferior	−0.065	0.546	0.798	−0.127	0.240	0.407
Nasal	−0.004	0.968	0.784	−0.176	0.101	0.176
Nasal-superior	−0.112	0.300	0.370	−0.129	0.233	0.294

PPA_+BM_ = β-parapapillary atrophy with Bruch’s membrane; PPA_−BM_ = β-parapapillary atrophy without Bruch’s membrane; D = diopters; IOP = intraocular pressure; BMO = Bruch’s membrane opening; LC = lamina cribrosa; FoBMO angle = angle of fovea-to-BMO-center axis relative to the horizontal axis of the image frame; BM = Bruch’s membrane; CT = choroidal thickness.

Values with statistical significance are shown in boldface.

^*^Standardized regression coefficient and *P* value adjusted for age and sex.

^†^*P* value adjusted for age, sex, and axial length.

All *P* values were adjusted with Benjamini-Hochberg procedure.

In multivariate linear regression model, to explore the independent factors associated with the microstructure of β-PPA, the PPA_+BM_ was negatively associated with LC thickness (*P* = 0.003) and temporal peripapillary CT (*P* = 0.034), and positively associated with anterior lamina depth (*P* < 0.001), FoBMO angle (*P* = 0.034), and macular BM length (*P* = 0.008). The overall *R*^*2*^ value derived from the multivariate linear regression model for PPA_+BM_ width was 0.472 (Table 3). As for PPA_−BM_, the multivariate model showed that only BMO area (*P* = 0.001) was independently associated with the PPA_−BM_ width in highly myopic eyes. The *R*^2^ value for the model was 0.247 (Table 4).

**Table 3:** Associations between enlargement of PPA_+BM_ width and ocular parameters in highly myopic eyes.

Variables	Multivariate analysis			
	Standardized regression coefficient	Unstandardized regression coefficient	95% CI	*P* value*
Axial length (mm)	0.151	35.173	−4.788 to 75.134	0.084
LC thickness (μm)	−0.283	−1.980	−3.173 to −0.786	**0.003**
Anterior laminar depth (μm)	0.323	0.631	0.305 to 0.957	**<0.001**
FoBMO angle (°)	0.190	10.391	1.077 to 19.704	**0.034**
Macular BM length (μm)	0.262	0.161	0.053 to 0.269	**0.008**
Temporal peripapillary CT (μm)	−0.191	−0.674	−1.280 to 0.069	**0.034**

PPA_+BM_ = β-parapapillary atrophy with bruch’s membrane; CI = confidence interval; LC = lamina cribrosa; FoBMO angle = angle of fovea-to-BMO-center axis relative to the horizontal axis of the image frame.

Values with statistical significance are shown in boldface.

*P values were adjusted with Benjamini-Hochberg procedure.

Factors showing multicollinearity were not included as explanatory variables.

Multivariate analysis included variables with *P* < 0.05 in univariate analysis.

**Table 4:** Associations between enlargement of PPA_−BM_ width and ocular parameters in highly myopic eyes.

Variables	Multivariate analysis			
	Standardized regression coefficient	Unstandardized regression coefficient	95% CI	*P* value*
Axial length (mm)	0.096	23.310	−24.139 to 70.758	0.331
BMO area (mm^2^)	0.348	103.645	43.105 to 164.186	0.001
Temporal peripapillary CT (μm)	−0.203	−0.739	−1.476 to 0.015	0.107

BMO = Bruch’s membrane opening; CT = choroidal thickness; CI = confidence interval.

Values with statistical significance are shown in boldface.

*P values were adjusted with Benjamini-Hochberg procedure.

Factors showing multicollinearity were not included as explanatory variables.

**Figure 2:**
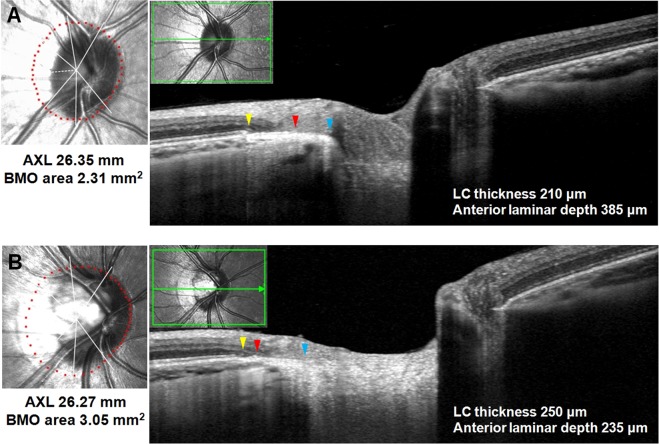
Images from myopic eyes with similar axial length and different microstructure of β-PPA. **(A)** A myopic eye of 29-year-old man with axial length of 26.35 mm. The width of PPA_+BM_ and PPA_−BM_ were 462 μm and 367 μm, respectively. The BMO area was 2.31 mm^2^. The EDI OCT image demonstrating a thinner LC and deeper anterior lamina surface compared with those from **B**. **(B)** A myopic eye 25-year-old man with axial length of 26.27 mm. The width of PPA_+BM_ and PPA_−BM_ were 157 μm and 485 μm, respectively. The BMO area was 3.05 mm^2^. The EDI OCT image demonstrating thicker LC thickness and shallower anterior lamina depth compared with those from **A**.

**Figure 3:**
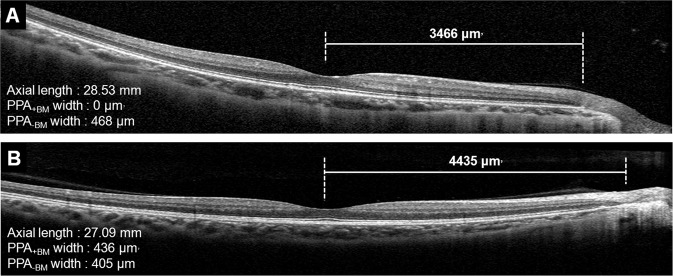
Representative cases showing relationship between the macular BM length and microstructure of β-PPA. **(A)** A myopic eye of 27-year-old-woman with PPA_−BM_ only. The macular BM length was 3466 μm. **(B)** A myopic eyes of 26-year-old woman with both PPA_+BM_ and PPA_−BM_. The macular BM length was 4435 μm. Although the eye of case A has longer axial length, the eye of case B has longer macular BM length. PPA_+BM_ = β-parapapillary atrophy with BM; PPA_−BM_ = β-parapapillary atrophy without BM.

We further analyzed the eyes with β-PPA (81 eyes) after dividing them into two groups according to the presence of PPA_+BM_: eyes with PPA_+BM_ group (n = 62) and eyes without PPA_+BM_ group (with PPA_−BM_ only; n = 19). Among 62 eyes with PPA_+BM_, 3 eyes had only PPA_+BM_, and 59 eyes had both PPA_+BM_ and PPA_−BM_. The mean width of PPA_+BM_ in 3 eyes having only PPA_+BM_ was 199.33 ± 9.71 μm. The mean width of PPA_+BM_ and PPA_−BM_ in 59 eyes with both PPA_+BM_ and PPA_−BM_ in their β-PPA area was 346.90 ± 160.26 μm and 415.51 ± 178.91 μm, respectively. In comparison with eyes with PPA_−BM_ only, those with PPA_+BM_ exhibited a significantly thinner LC (*P* = 0.009), deeper anterior lamina surface (*P* = 0.029), larger FoBMO angle (*P* = 0.007), longer macular BM length (*P* = 0.010), and thinner subfoveal CT (*P* = 0.037) (Table 5). Figures 2 and 3 present representative cases of highly myopic eyes with a different microstructure of β-PPA.

**Table 5:** Comparison of characteristics between highly myopic eyes with PPA_+BM_ and without PPA_+BM_ (with PPA_−BM_ only).

Variables	Eyes with PPA_+BM_ (n = 62)	Eyes with PPA_−BM_ only (n = 19)	*P* value*
IOP (mmHg)	14.00 ± 2.32	13.58 ± 2.65	0.509
Axial length (mm)	27.11 ± 0.94	26.91 ± 0.77	0.397
BMO area (mm^2^)	2.75 ± 0.69	2.68 ± 0.85	0.720
PPA_+BM_ width (μm)	339.76 ± 159.51	0	**<0.001**
PPA_−BM_ width (μm)	395.40 ± 196.25	400.10 ± 189.11	0.852
LC thickness (μm)	182.82 ± 30.29	202.79 ± 31.23	**0.009**
Anterior laminar depth (μm)	346.97 ± 109.29	293.84 ± 93.05	**0.029**
FoBMO angle (°)	−4.50 ± 3.66	−6.02 ± 4.22	**0.007**
Macular BM length (μm)	4121.66 ± 321.21	3894.79 ± 350.03	**0.010**
Presence of LC defect, n (%)	29 (46.77)	9 (47.37)	0.995
Subfoveal CT (μm)	221.40 ± 85.76	250.74 ± 93.02	**0.037**
**Peripapillary CT (μm)**			
Global	143.41 ± 42.48	135.63 ± 47.51	0.500
Temporal-superior	153.46 ± 50.48	153.63 ± 65.42	0.990
Temporal	123.20 ± 50.34	135.37 ± 66.93	0.399
Temporal-inferior	112.13 ± 41.56	113.00 ± 44.85	0.938
Nasal-inferior	125.33 ± 41.10	113.21 ± 40.28	0.263
Nasal	164.56 ± 45.94	136.63 ± 38.35	0.065
Nasal-superior	170.10 ± 50.17	161.16 ± 51.67	0.503

IOP = intraocular pressure; BMO = Bruch’s membrane opening; PPA_+BM_ = β-parapapillary atrophy with Bruch’s membrane; PPA_−BM_ = β-parapapillary atrophy without Bruch’s membrane; LC = lamina cribrosa; FoBMO angle = angle of fovea-to-BMO-center axis relative to the horizontal axis of the image frame; BM = Bruch’s membrane; CT = choroidal thickness.

Values with statistical significance are shown in boldface.

*P values derived from Student’s t-test in normally distributed continuous variables, Mann-Whitney U test in non-normally distributed continuous variables, and chi-square test in categorical variable.

## Discussion

In this study, we evaluated 89 highly myopic eyes with axial length greater than 26 mm, and β-PPA was found in 81 of 89 eyes (91.01%). The microstructure of β-PPA was different among the 81 eyes; 19 eyes had only PPA_−BM_, whereas 62 eyes had PPA_+BM_ in their β-PPA area. Importantly, we observed that anatomical parameters associated with the width of PPA_+BM_ and PPA_−BM_ were markedly different, after adjusting for significant confounders, age, sex, and axial length^2,3^. The large PPA_+BM_ was associated with a thin LC, deep anterior lamina surface, long macular BM length, close FoBMO axis relative to the horizontal axis, and temporal peripapillary CT thinning. Meanwhile, the width of PPA_−BM_ was positively associated with BMO area. To our knowledge, this is the first work thoroughly investigating the ONH characteristics which have a different β-PPA microstructure among highly myopic subjects. Earlier researches cannot be compared with the current study since earlier works included a wide range of age and refractive error groups^2,3,20^. Our conclusions are clinically important given the importance of β-PPA in myopic eyes, and furthermore, this research might provide clues to a association between PPA_+BM_ and glaucoma susceptibility in myopic eyes.

PPA_−BM_, sometimes termed as the gamma zone, is thought to be the result from stretching of peripapillary border tissue during globe elongation. The strong association between axial length and PPA_−BM_ width has been well documented^1–3,5,20,21^. However, this relationship seems to be non-linear in clinical practice, and Jonas *et al*.^1^ described the prevalence and size of PPA_−BM_ increased steeply, starting at an axial length of 26.5 mm, in their histomorphometric investigations of human globes. Recently, Zhang *et al*.^20,21^ demonstrated a steep increase in BMO diameters beyond an axial length of 26.0 mm and reported that such BMO enlargement was significantly associated with the large PPA_−BM_ in myopic eyes. Similarly, we found a significant positive association between the PPA_−BM_ width and BMO area, after adjusting for significant confounders. Following the speculation by Zhang *et al*.^20^, we suggest that development and enlargement of the PPA_−BM_ may be due to the disproportional enlargement between BMO and the peripapillary sclera opening. When the BMO enlarges during axial elongation, the peripapillary sclera opening might also enlarge, but due to the LC anchored at the peripapillary sclera flange, the peripapillary sclera opening enlargement cannot keep pace with the BMO enlargement. Regarding the reason for the location of PPA_−BM_ (most PPA_−BM_ is found at the temporal side of the ONH), possibly the concomitant nasal shifting of LC might cause the asymmetric stretching of nasal and temporal peripapillary border tissue, thus resulting in enlargement of the PPA_−BM_ mostly at the temporal side of the ONH. Recently, Kim *et al*.^7,22,23^ introduced the concept of LC nasal shifting during myopia progression after the prospective longitudinal observation of the ONH in South Korean children. However, this concept is only a hypothesis and it has not been scientifically confirmed, thus future longitudinal investigations might be needed to confirm our assumption. Meanwhile, the BMO area was not associated with the width of PPA_+BM_, which implies that enlargement of PPA_−BM_ and PPA_+BM_ might be the result of different mechanisms during axial elongation.

It has been suggested that PPA_+BM_ results from age-related degeneration of the RPE-BM complex. Curcio *et al*.^4^ previously demonstrated that the length of BM bared of RPE cells was significantly wider in the oldest eyes in their histomorphometric study. However, we have shown that PPA_+BM_ can also be found in young myopic eyes. Our results are in line with recent reports^6,7^. The mechanism of RPE atrophy in the peripapillary region with aging is not fully determined. It is generally thought to be caused by insufficient blood supply due to a thinner choroid^24,25^. Regarding the PPA_+BM_ found in young myopic eyes, Lee *et al*.^7^ postulated that RPE migration over the BM or the BM stretching, rather than the ischemic insults, might be associated with the PPA_+BM_ enlargement during axial elongation. They speculated that mechanical stress on the peripapillary area during axial elongation might be involved in the enlargement of the PPA_+BM_ in highly myopic eyes. Our result, which shows a significant positive relationship between the macular BM length and PPA_+BM_ width, might support their speculation.

Jonas *et al*.^17^ showed that BM length in the macular lesion was not affected by axial elongation. They suggested that the axial elongation-related increase in the optic disc-fovea distance predominantly occurred through an enlargement of PPA_−BM_, whereas the macular BM does not expand. In line with their findings, there was no significant relationship between the axial length and macular BM length (*r* = −0.100, *P* = 0.351) in this study. Interestingly, we observed a statistically significant relationship between the macular BM length and the PPA_+BM_ width among highly myopic eyes in multivariate analysis. Our findings might indicate that BM can be stretched during axial elongation, which may affect PPA_+BM_ enlargement in highly myopic eyes. The close relationship between the FoBMO angle and PPA_+BM_ width might be explained by macular BM stretching, since the FoBMO axis relative to the ONH could be affected by the degree and direction of macular BM stretching.

Interestingly, we observed that eyes with large PPA_+BM_ represent characteristic structural changes in LC. The larger PPA_+BM_ was significantly related to a thinner LC and deeper anterior lamina surface. Previous investigations revealed LC thinning in highly myopic eyes, and it was speculated that axial elongation-associated thinning of the LC might be due to enlargement of BMO^26^. However, we could not found a significant correlation between the LC thickness and BMO area (*r* = 0.089, *P* = 0.406) in our highly myopic subjects. Our finding shows that the ONH biomechanics are complex in myopic eyes, and we believe that the individual biomechanical properties of LC and adjacent peripapillary tissue might contribute to this complexity. Further investigation is needed to clarify the mechanism of LC changes found in eyes with a large PPA_+BM_.

It is well known that the choroid is markedly thinner in highly myopic eyes than in emmetropic eyes^27,28^. Fang *et al*.^29^ demonstrated that the choroidal change does not occur symmetrically and that there is a sequence for thinning. They suggested that this progressive and continuous choroidal thinning plays a crucial role in the development of various degrees of myopic maculopathy. Based on their observation, choroidal thinning starts from the temporal peripapillary region to the fovea and then enlarges toward the entire posterior pole. Considering that the choroidal thinning in high myopia is mechanical strain-dependent, such a non-uniform thinning of the choroid suggests that there might be an asymmetry in ocular growth patterns during myopia progression. Also, it indicates that temporal peripapillary and subfoveal choroid regions are more susceptible to mechanical strain than the other regions in myopic eyes. In line with their findings, in our study, both subfoveal and temporal peripapillary CT showed a tendency for thinning as the width of PPA_+BM_ and PPA_−BM_ became larger. However, the thinning was more remarkable in eyes with large PPA_+BM_. In multivariate analysis, temporal peripapillary CT was significantly associated with the width of PPA_+BM_, and eyes with PPA_+BM_ exhibited significantly thinner subfoveal CT compared with eyes with PPA_−BM_ only. Our findings suggest that eyes with large PPA_+BM_ might have undergone more strain on the region between the fovea and the ONH during eyeball growth. Moreover, this hypothesis would be supported by macular BM lengthening in eyes with large PPA_+BM_.

Combining all these findings, we propose that the differential development of β-PPA in highly myopic eyes might result from different degrees of mechanical strain and their topographical variation during axial elongation. Large BMO area was associated with the large PPA_−BM_. Whereas large PPA_+BM_ was associated with the longer macular BM, thinner subfoveal and peripapillary choroid, and some characteristic LC changes. If the mechanical strain is too high thus BMO widening is not enough to release this strain, the LC might be affected (thinning and deepening) and subsequently, BM lengthening and subfoveal choroid thinning might occur. These anatomical changes might be associated with the individual difference in biomechanical property of BM during axial elongation.

Limitations of our study include the modest sample size. However, by employing strict inclusion criteria regarding age and axial length, we could meticulously exclude the influence of age-related degeneration of the parapapillary area and investigate the exclusive influence of myopia. Another limitation is related to the difficulties of imaging BMO and deep ONH structures. The determination of BMO might be inaccurate in highly myopic eyes, and the anterior and posterior lamina surface could not be visualized in some healthy eyes^30^. However, in this study, we strictly excluded such patients having inadequate visibility of BMO or LC (11.88%). Third, the anterior laminar depth was measured at the center of the ONH. We did not use the BMO center as measurement location, because BMO center is often close to the disc edge in the tilted optic disc and, thus, considered to be not suitable for measuring anterior laminar depth. However, detection of ONH center by IR fundus image is sometimes difficult in myopic eyes. There is a possibility that the uncertain location of the ONH center might have caused inaccurate anterior laminar depth measurement. Fourth, the associations between the width of PPA_+BM_ and LC changes could be affected by IOP, since LC is highly sensitive to IOP. However, the relationships between the width of PPA_+BM_ and LC thickness and anterior laminar depth were remained statistically significant after adjusting for age, sex, axial length, and IOP (β = −0.402, *P* < 0.001 and β = 0.285, *P* = 0.008, respectively), thus the IOP is not likely to affect the main outcome of our study. Finally, this study is cross-sectional. Therefore, it is uncertain whether the previously described characteristics are the results of axial elongation. For example, Fang *et al*.^29^ reported a progressive choroidal thinning with aging in young, highly myopic adults (4.91 μ/year). The subfoveal and peripapillary choroidal thinning found in eyes with enlarged PPA_+BM_ could be a result of the aging process, not a result of axial elongation induced mechanical strain. A future longitudinal study could help to clarify this study’s findings.

In conclusion, we have documented structural differences in the ONH and posterior pole in highly myopic eyes with a different β-PPA microstructure. Large PPA_+BM_ was associated with the longer macular BM and structural changes in the LC. Future studies might be needed to elucidate the association between these structural features and development of glaucoma in myopic eyes.

## Data Availability

The datasets generated during the current study are available from the corresponding author on reasonable request.
